# Hemorrhoidal disease among doctors from grade-A tertiary hospitals in big cities of China: results from web-based doctors as patients survey

**DOI:** 10.1186/s12876-024-03166-2

**Published:** 2024-03-13

**Authors:** Xiaoyuan Qiu, Yuxin Liu, Weikun Shi, Guole Lin, Mei Rong, Bingjie Wang

**Affiliations:** 1 Chinese Academy of Medical Sciences and Peking Union Medical College, Beijing, 100730 China; 2 Department of Surgery, Peking Union Medical College Hospital, (Dongdan Campus), No.1 Shuaifuyuan Wangfujing Dongcheng District, Beijing, 100730 China; 3 Medical & Scientific Affairs, Servier, Beijing, 100020 China

**Keywords:** Doctors, Hemorrhoidal disease, MPFF, Oral medication, Survey

## Abstract

**Background:**

Doctors are at high risk of developing hemorrhoidal disease (HD), but it is unclear whether doctors are aware of this risk. The OASIS (dOctors AS patIentS) study was performed to examine the prevalence, awareness, diagnosis, and treatment of HD among doctors in big cities in China.

**Methods:**

An online survey consisting of a structured questionnaire was carried out among doctors in grade-A tertiary hospitals in 29 provinces across China from August to October 2020.

**Results:**

A total of 1227 questionnaire responses were collected. HD prevalence was 56.8%, with a significant difference between internists and surgeons (*P* = 0.01). 15.6% of doctors with HD didn’t have serious concerns about the recurrence and severity of HD. 91.5% of doctors adopted general treatments, and 83.0% considered oral medications only when topical medications were ineffective. Among the oral medications, Micronized Purified Flavonoid Fraction (MPFF) was most effective based on the scores from three important parameters, but only 17% of doctors received MPFF.

**Conclusions:**

Doctors are at higher risk of developing HD with a high prevalence among Chinese doctors, but they are not fully aware or not concerned about HD. There is a deficiency in treatment recommendations and clinical management of HD even for doctors, including late initiation and inadequate oral drug therapy. Therefore, awareness and standardized treatment of HD should be improved among Chinese doctors, as well as in the general population.

**Supplementary Information:**

The online version contains supplementary material available at 10.1186/s12876-024-03166-2.

## Background

Hemorrhoidal disease (HD) is a common anorectal disease [1], generally characterized by bleeding, swelling, prolapse, pain, itching, and anal discomfort of the hemorrhoids. In HD, the blood is bright red and not mixed with feces, but instead coats the feces or drops after a bowel movement. Severe grade HD not only affects the patient’s quality of life but also requires urgent hospitalization and blood transfusion due to massive hemorrhage [2].

A precise medical history and thorough physical examination, including digital rectal examination and anoscopy, are imperative for the diagnosis of HD. Unless bright red blood is observed from hemorrhoids, any patients with rectal bleeding should be examined by flexible sigmoidoscopy or colonoscopy, especially individuals at risk of colorectal cancer [3]. However, probably because of common and inconspicuous symptoms at disease onset, patients tend to use self-medication rather than seek proper medical treatment.

Treatment options for HD range from conservative management with diet, physical activity, training in toilet use, and topical or oral medications, to surgical interventions. Nonsurgical outpatient procedures are also available, including rubber band ligation, infrared coagulation, and sclerotherapy. The best treatment option for the various grades of this disease remains debatable; however, a guiding principle is to carry out less invasive options first.

Many risk factors for HD are known, including age, gender, pregnancy, dietary and bowel habits. [3–6]. The predisposing factors of symptomatic hemorrhoids include elevated intra-abdominal pressure, e.g., increased pressure due to pregnancy and straining. Other contributing factors are weakening of the supporting connective tissue, smooth muscle, and vasculature from advancing age as well as activities such as strenuous lifting, straining with defecation, and prolonged sitting [7, 8].

Some studies have established specific treatment guidelines for pregnant women and elderly adults with HD [3]. However, profession is also one of the most important factors [9]. Doctors’ work habits, e.g., high stress at work, inadequate sleep, and long standing and sitting working positions, put them at higher risk of developing HD. However, doctors are not normally considered patients and no systematic study has assessed the prevalence of HD among Chinese doctors. Thus, we designed this “doctors as patients” survey-based study to examine the prevalence, diagnosis, and treatment of HD among doctors in different grade-A tertiary hospitals in China. We aimed to consider mild to severe symptoms of HD to find the best treatment options.

## Methods

### Subjects and survey design

A web-based voluntary survey conducted by a third-party platform in China (http://www.medlive.cn/), which enrolled nearly 1,340,000 registered doctors (License required for registration) is available. The survey is a convenient sample of Chinese doctors, and sample quotas were designed based on the principle of stratified equal probability sampling according to the registered doctors. Participants were recruited from August to October 2020. Email and SMS communications were used, and doctors clicked on the sent link to participate in the online survey. To avoid multiple responses by a single person, each IP address was set to answer only once. The questionnaire was designed by well-recognized experts in chronic venous diseases, ahead of sampling. The online questionnaire was then programmed, and a small number of doctors were recruited for validation; a comprehensive recruitment was initiated after the examination was correct. The online questionnaire had two main parts, including the screening and main questionnaires. The main questionnaire was comprised by general information and HD treatment experience (Supplementary materials).

The screening questionnaire was designed to identify doctors with attending or above level based on Grade-A tertiary (highest level) hospitals from big cities (3rd-tier or above, according to 2019 Ranking of Cities’ Business Attractiveness) of China and used to investigate HD prevalence and comorbidities. The main questionnaire could only be filled by those who passed the screening questionnaire. The main questionnaire was deigned to further record personal information and diagnosis, disease status and understanding, treatment options and preferences for oral medications in HD. The collected responses were checked for errors such as omissions and logically unseasonable answers. Descriptive data analysis was performed of the cleaned dataset after quality control.

### Consent to participate

Before starting the survey, participants were required to consent to sharing some medical and health information about themselves. In case of no consent, the survey was stopped. Participant data were used in a general and anonymized manner for this study only. No data were provided to any third parties.

### Statistical approach

Relevant data were recorded in Excel sheets and statistically analyzed with SPSS 21.0. The Chi-square test was used to determine statistical significance for each dimension of categorical variables (multiple choice questions), and the F test was used for continuous variables (numeric entry questions). In the test of significance, both the chi-square and F tests specified that at the 95% confidence level, differences in variables and dimensional groups were considered significant with *P* < 0.05. In case of skipping some questions due to logical relationship or a respondent’s omission, such data were included in the analysis if the overall data was not affected, and the sample size was separately marked.

## Results

The survey involved 342 grade A hospitals, with the geographical coverage of 29 provinces (autonomous regions and municipalities) in China. The doctors included had attending and above professional titles in internal medicine and surgery, including general medicine, cardiology, respiratory medicine, gastroenterology, neurology, medical oncology, endocrinology, infectious disease, nephrology, general surgery, orthopedics, urology, obstetrics and gynecology, hepatobiliary surgery, breast surgery, neurosurgery and vascular surgery, etc. A total of 23,630 doctors were sent the questionnaire by email or SMS. The number of clicks was 4,077 (a click-through rate of 4077/23,630 = 17.3%), of which 2,850 people did not meet the above inclusion criteria (2,651 individuals due to unwillingness to participate, 72 due to city level, 112 due to hospital level and 15 due to professional title). Finally, 1227 individuals passed the screening, indicating a pass rate of 1227/4077 = 30.1%.

### HD prevalence and comorbidities

Among the 1,227 included doctors, 697 had HD, i.e., a prevalence of 56.8%. There were 621 internists and 606 surgeons in this survey, with HD prevalence rates of 60.4% (375/621) and 53.1% (322/606) respectively, indicating a statistically significant difference between internists and surgeons (*P* = 0.01). Among the doctors with HD, 39.7% (277/697) had cervical or lumbar spine disease, 32.3% (225/697) had sleep disorder, 26.1% (182/697) had chronic venous disease, 22.5% (157/697) had chronic gastritis or gastric ulcers, 18.4% (128/697) had hypertension and 10.3% (72/697) had urinary calculus.

### General information of the doctors with and without HD

After passing the screening questionnaire, 331 doctors successfully completed the main questionnaire. Among them, 212 doctors with HD completed all the contents of the main questionnaire, and 119 doctors without HD only completed the general information contents in the main questionnaire. The general geographic distribution well represented the regional balance of registered doctors.

Among the 212 doctors with HD, the duration of HD ranged from 1 to 35 years, averaging 8.9 years. They included 59.4% (126/212) men and 40.6% (86/212) women. Patient age ranged from 29 to 70 years, averaging 43.6 years. Totally 76.9% (163/212) exerted internal medicine. Among the 119 doctors without HD, 63.0% (75/119) were male and 37.0% (44/119) were female. Patient age ranged from 31 to 63 years, averaging 44.5 years. The majority of these doctors exerted internal medicine, accounting for 85.7% (102/119).

Compared with doctors without HD, those with HD had significantly longer defecation time (defecation lasting more than 10 min/time; 20.8% [44/212] VS 9.2% [11/119], *p* = 0.006). Besides, female doctors with HD had increased pregnancy times (previous pregnancy 2 or more; 30.2% [26/86] VS 25.0% (11/44]), smoking (10.4% [22/212] VS 8.4% [10/119]), and wrinkled, lumpy or nut-like defecation shape (32.1% [68/212] VS 18.5% [22/119]), but these differences were not significant (Table 1).

**Table 1:** Clinical characteristics of the examined doctors with and without HD

Parameter	Doctors with HD *n* = 212	Doctors without HD *n* = 119	*P* value
Age (Years)	43.6	44.5	0.321
Gender (male, n, %)	126, 59.4	75, 63	0.521
BMI	23.7	23.7	0.998
Sitting time > 10 h per week (%)	78.8	79.0	0.662
Exercise time < 0.5 h per week (%)	17.9	13.4	0.740
Proportion of “sitting” working state (%)	54.9	55.0	0.975
Smoking (%)	10.4	8.4	0.560
Number of defecations (times)	1.1	1.3	0.395
Defecation time (> 10 min, %)	20.8	9.2	0.006
Defecation shape (wrinkled, lumpy or nuts-like, %)	32.1	18.5	0.144
Female doctors with previous pregnancy (2 or more, %)	30.2 (26/86)	25.0 (11/44)	0.532

#### Conditions of HD

**Fig. 1:**
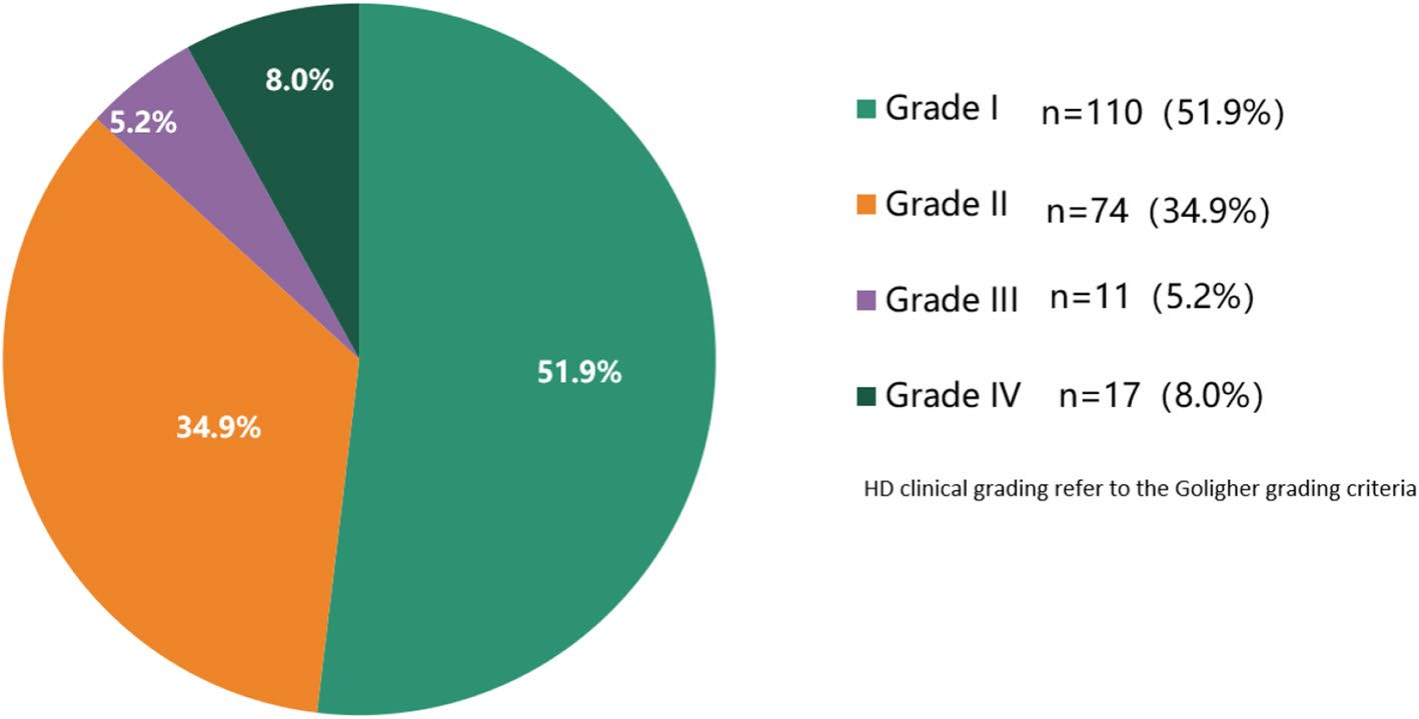
Clinical grades of HD among doctors

Among the 212 doctors with HD, 51.9% (110/212) had clinical grade I HD, 48.1% (102/212) had grade II and above HD (referred to the Goligher’s grading criteria^7^) (Fig. 1). Doctors with severe HD were relatively older (mean ages of doctors with grade I, II, and III&IV disease were 42.6, 43.8 and 47.3 years, respectively; *P* = 0.014). Besides, the higher the HD grade, the longer the sitting time of the doctors: 73.6% (81/110), 82.4% (61/74) and 89.3% (25/28) of doctors with grade I, II and III&IV disease had sitting time > 10 h per week, respectively. In addition, the higher the HD grade, the shorter the exercise time: 13.6% (15/110), 21.6% (16/74) and 25% (7/28) of doctors with grade I, II and III&IV disease had exercise time < 0.5 h per week, respectively). However, the above differences were not statistically significant. What’s more, the higher the HD grade, the higher the proportion of females who suffered: the proportions of females with grade I, II and III&IV disease were 24.5% (27/110), 56.8% (42/74) and 60.7% (17/28), respectively (*P* = 0.000) (Table 2).

**Table 2:** Characteristics of doctors with different clinical grades of HD

Parameter	Clinical grade of HD			*P* value
	Grade I *n* = 110	Grade II *n* = 74	Grade III & IV *n* = 28	
Age (Years)	42.6	43.8	47.3	0.014
Gender (male, %)	75.5	43.2	39.3	0.000
BMI	24.2	23.0	23.8	0.011
Sitting time > 10 h per week (%)	73.6	82.4	89.3	0.598
Exercise time < 0.5 h per week (%)	13.6	21.6	25.0	0.569
Proportion of “sitting” working state (%)	56.7	52.7	53.6	0.442
Smoking (%)	88.2	89.2	96.4	0.437
Number of defecations (times per day)	1.2	0.9	1.0	0.584
Defecation time (> 10 min each time, %)	17.3	24.4	25	0.509
Defecation shape (wrinkled, lumpy or nuts-like, %)	55.4	62.2	60.7	0.255
Female doctors with Previous pregnancy (2 or more, %)	29.6 (8/27)	28.6 (12/42)	35.3 (6/17)	0.875

**Fig. 2:**
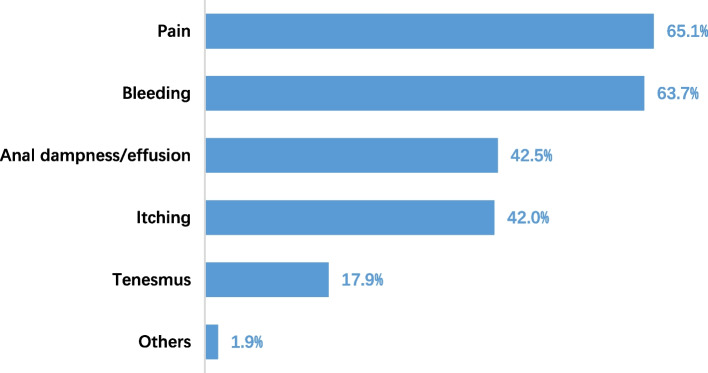
The most troublesome symptoms

Among the 212 doctors with HD, the most bothersome symptoms were pain (65.1%, 138/212), followed by bleeding (63.7%, 135/212) (Fig. 2). The participants stated that the impact of HD on their quality of life was moderate, with an average score of 4.1 (1 to 10, with 1 indicating little impact and 10 indicating extremely large impact; the higher the score, the lower the quality of life). About 31% of doctors stated that they were bothered with the disease more than 20% of the time per month, i.e., about 6 days per month. The higher the grade of HD, the more the impact on life. The average scores for grade I, II and III&IV HD were 3.4, 4.6 and 5.5, respectively (*p* = 0.000); 18.2% (20/110), 41.9% (31/74) and 46.4% (13/28) of doctors were bothered more than 20% of the time each month with grade I, II and III&IV HD, respectively (*p* = 0.001) (Table 3).

**Table 3:** Impact of HD on doctors by clinical grade

Bothered time per month	Clinical grade of HD			*P* value
	Grade I	Grade II	Grade III and above	
< 20%	81.8%	58.1%	53.6%	0.001
20%-40%	16.4%	33.8%	35.7%	
40%-60%	1.8%	6.8%	3.6%	
> 80%	0.0%	1.4%	7.1%	

#### Diagnosis and treatment of HD

In terms of HD knowledge, 15.6% (33/212) of doctors with HD stated that they did not know “HD is one of the venous diseases and often complicated with chronic venous disease (commonly known as varicose veins)”. The difference in this aspect among doctors with various professional titles was statistically significant: the proportions among attending doctors, chief doctors and associate chief doctors were 26.4% (14/53), 14.4% (15/104) and 7.3% (4/55), respectively (*P* = 0.021).

**Fig. 3:**
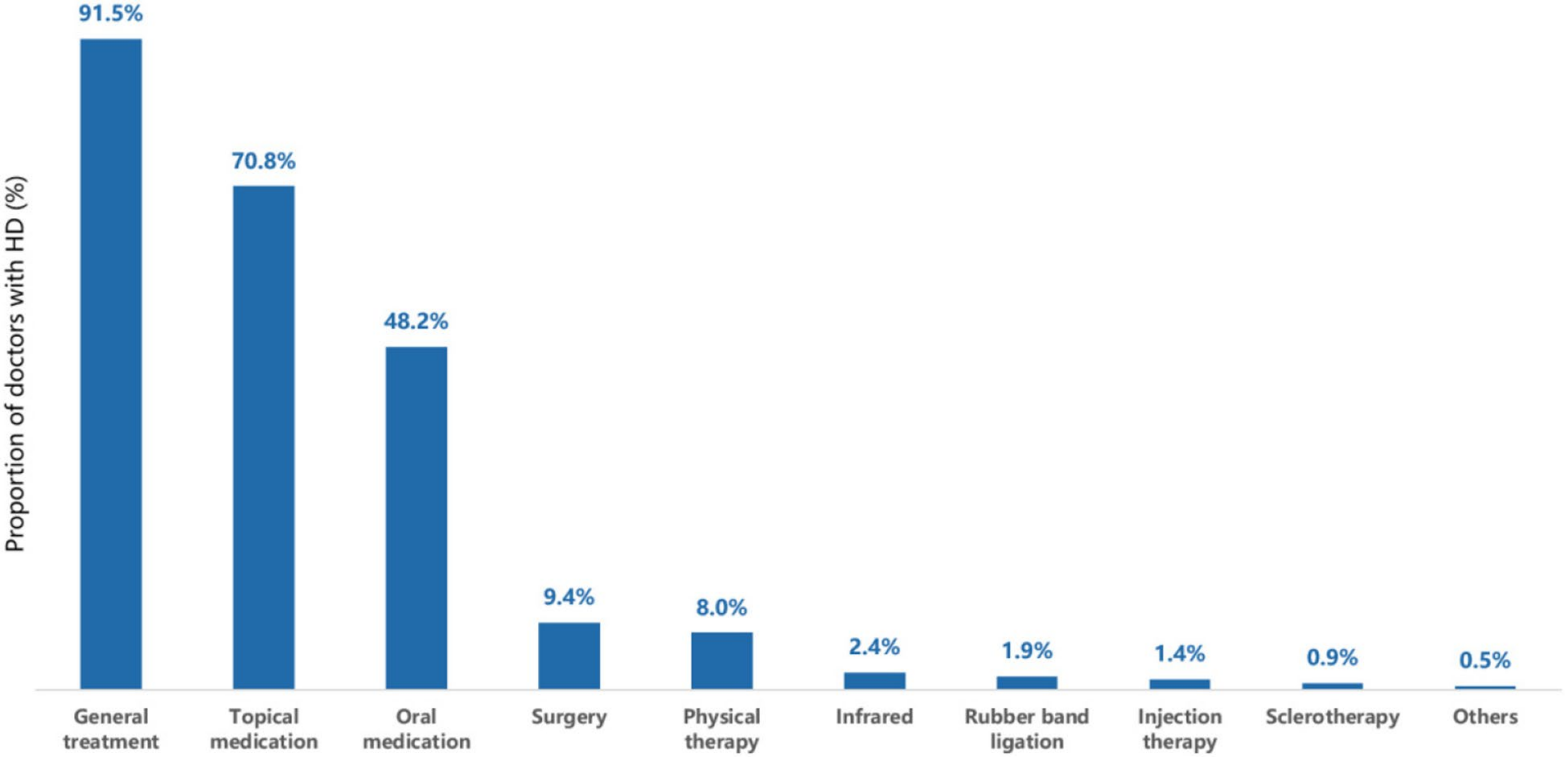
Treatment choices for doctors with HD. General treatment: more frequent intake of fruits and vegetables and water, change of bowel habits, maintaining smooth defecation, avoidance of sitting or standing for a long time, and appropriate exercise, etc.

When it comes to diagnosis, 50.5% (107/212) of doctors with HD were self-diagnosed, 39.6% (84/212) were diagnosed by general or anorectal specialists and 9.0% (19/212) were diagnosed by physical examination and 0.9% (2/212) by other diagnostic methods. All doctors with HD underwent treatments, of which the most was general treatment (91.5%, 194/212), mainly consisting of lifestyle and bowel habit changes, followed by topical medication (70.8%, 150/212) and oral medication (48.2%, 102/212) (Fig. 3). For treatment efficacy, 25.0% (53/212) of doctors considered that the treatment was effective, and the disease was not recurrent, while 71.1% (151/212) considered that despite the effective treatment HD could be recurrent, and 3.3% (7/212) considered that the treatment was ineffective.

**Fig. 4:**
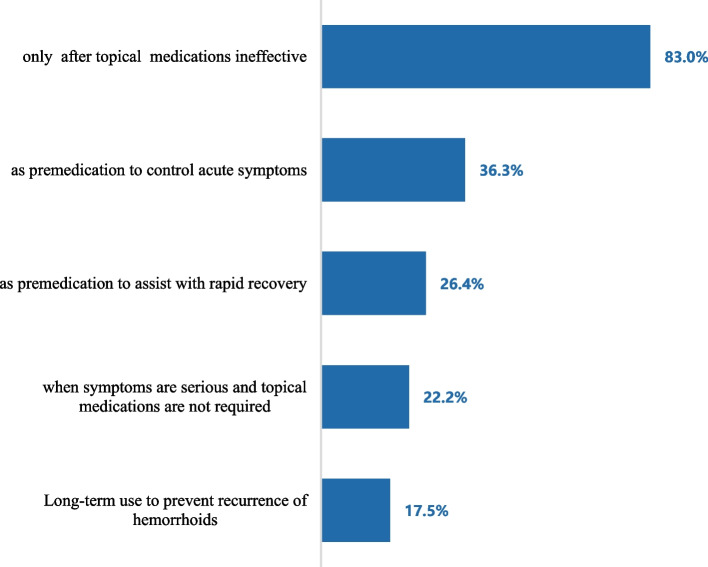
Timing of initiating oral medication

Aescuven forte (Aescuven forte Tablets, Maizhiling; 34.4%, 73/212), MaYingLong (Diosmin Tablets; 32.1%, 68/212) and Alvenor (Micronised Purified Flavonoid Fraction, MPFF, Citrus Bioflavonoids Tablets; 17.0%, 36/212) were the top 3 oral medications commonly used. In terms of timing of using oral medications, most of the doctors with HD (83.0%, 176/212) considered that “oral medications are used only after ineffective topical medications”, followed by “oral medications can be used as premedication to control acute symptoms” (36.3%, 77/212) and “oral medications can be used as premedication to promote rapid recovery” (26.4%, 56/212) (Fig. 4).

**Fig. 5:**
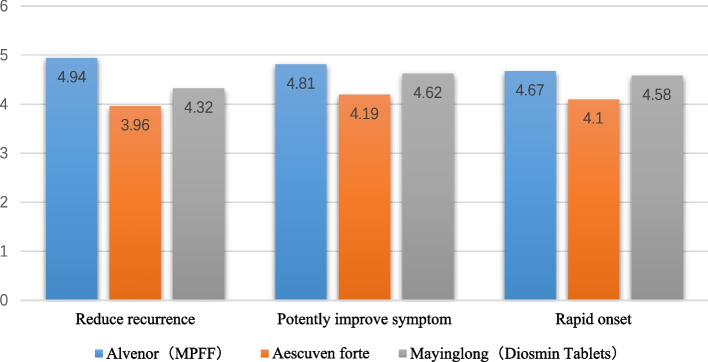
Performance scores for the top 3 commonly used oral medicines the doctors valued most. Score1-7, with 1 indicating totally disagree and 7 indicating totally agree

When choosing oral medications, doctors attached the most importance to “reduce recurrence” (55.7%, 118/212), “potently improve symptoms” (48.6%, 103/212) and “rapid onset” (48.1%, 102/212). Doctors also believed that oral medications can “have better efficacy in combination with topical medications” (36.8%, 78/212), “directly act on the core of the disease by the mechanism of action” (36.8%, 78/212), “be safer as they are extracted from natural plants” (28.8%, 61/212) and “be used as premedication to rapidly control acute symptoms before surgery and accelerate postoperative recovery” (11.8%, 25/212). Doctors scored the above medical performance characteristics of Mayinglong, Aescuwen and MPFF, and MPFF performed best in all three aspects, while Aescuwen had the lowest score (Fig. 5).

## Discussions

HD is one of the commonest anorectal diseases in China. The latest nationwide epidemiological survey showed that the prevalence of HD in China is 50.28% [10]. The results reported by a population survey in different regions of China are similar to the above conclusions [2]. Further subgroup analysis revealed that besides dietary habits and bowel habits, occupation is also an important influencing factor in HD [11], and professionals with stressful work, irregular life, inadequate sleep and need to be sedentary are at higher risk of developing HD [12]. Internal hemorrhoids are generally graded based on the Goligher’s classification: Grade I, non-prolapsing hemorrhoids; Grade II, prolapsing hemorrhoids on straining but reduce spontaneously; Grade III, prolapsing hemorrhoids requiring manual reduction; Grade IV, non-reducible prolapsing hemorrhoids [13].

Individuals with sedentary working habits are of particularly higher risk. On the one hand, long-term exposure to a fixed posture affects blood circulation leading to pelvic blood stasis and venous congestion, which greatly affects digestion, absorption and the peristaltic function of the intestine, causing constipation and HD [11]. On the other hand, the sedentary state leads to autonomic dysfunction and perineal dampness, resulting in inflammation that induces HD and other anorectal diseases [9]. The occupational characteristics of doctors accord with a high risk of HD, e.g., stressful work, irregular life, inadequate sleep and strenuous activity, causing an excessive increase of intra-abdominal pressure [9, 10], making them a high-risk group for the disease. HD prevalence was about 56.8% among doctors in grade-A tertiary hospitals in this study, which was significantly higher than that of the general population. The internists had a significantly higher prevalence of HD than surgeons (60.4% vs.53.1%, *p* = 0.01). This may be related to the need for internists to adopt the "sitting" position for longer periods at work. As shown above, the longer the doctors sit, the higher the HD grade. Pregnancy also puts pressure on the veins in the lower body, making female doctors show higher incidence of higher-grade HD (a proportion of females with grade III&IV of 60.7%).

In addition, HD and chronic venous disease have a common etiology and similar risk factors. Low-fiber diet induces chronic constipation, which increases intra-abdominal pressure. It was suggested that this pressure would easily be transmitted to the hemorrhoidal plexus that has no valves. The valves of the lower limb veins provide initial protection but eventually become incompetent and expose the veins to high pressure [14, 15]. The multinational CHORUS study [16] showed a significant correlation between HD and chronic venous disease (*P* = 0.004). This corroborates the current study in which 26.1% of the examined doctors with HD had concomitant chronic venous disease, a rate higher than the prevalence of chronic venous disease in the general population in China (8.89%) [17]. The population of this study included doctors with strong medical background; however, HD awareness was still disappointing: 15.6% of doctors with HD stated they did not know that “HD is one of the venous diseases and often complicated with chronic venous disease”.

Symptoms attributed to hemorrhoids encompass bleeding, pain, pruritus, fecal seepage, prolapse and mucus discharge [18, 19]. Doctors considered the most bothersome symptoms include pain and bleeding (65.1% and 63.7%, respectively). That might be because doctors relatively seldom panic about bleeding owning to their profession, while pain haunts them almost every minute. When it comes to the treatments administered, multiple patients tend to use self-medication rather than to seek proper medical attention [20], so do doctors. More than 50% of doctors with HD in this study were self-diagnosed. From our point of view, bleeding or anal pain should not be diagnosed as hemorrhoids until the colon is adequately examined. Thus, as soon as a symptom occurs, individuals should consult general or anorectal specialists.

When a provisional diagnosis of HD is made, basic treatment can be started, such as sufficient water intake, healthy diet, physical activity and toilet training [11, 21]. Although almost all doctors received general treatment, the effect was unsatisfactory, and 71.7% stated that although effective, HD was recurrent; this self-reported recurrence rate is much higher than that of a recent systematic review (71.7% vs 56.5%) [22], which may be attributed to non-standard treatment. Only 48.2% of doctors used oral treatment, but most of them only used oral medications after failure of topical drugs or in case of very severe disease.

Actually, the main goal of medical treatment is to control the acute symptoms of hemorrhoids. Guidelines for HD treatment in China and worldwide recommend oral veno-active drugs as the reference treatment that can be administered to patients with all clinical grades and relieve symptoms such as pain, bleeding, etc., preventing recurrence [2, 21, 23]. The latest 2020 China HD guideline recommends MPFF as the preferred veno-active drug for the clinical treatment of grade I-IV HD (level 1A recommendation). It also recommends MPFF as an adjunct treatment to device and surgical therapy (level 1A recommendation), and MPFF or non-steroidal anti-inflammatory drugs and topical agents containing aluminum sulfate as an adjunct in HD cases for the improvement of postoperative symptoms (level 1A recommendation) [2]. Subgroup analysis in the multinational CHORUS study showed that the utilization rate of intravenous active drugs in HD cases is as high as 95.7%, with 86.2% selecting MPFF among veno-active drugs [16]. However, the current study indicated that although MPFF has shown superiority in satisfying doctor-valued benefits, its usage rate was only 17%, which is much lower than the application rate of MPFF in HD cases generally.

This study reported a comprehensive analysis of HD awareness, diagnosis and treatment in the examined professional group (doctors with attending or above level in Grade-A tertiary hospitals from big cities). Indeed, as medical professionals, doctors should be significantly better than the general population in disease prevention, self-diagnosis, and self-treatment. However, in the present study, doctors still had significant cognitive deficiencies regarding the common "minor disease" of HD. This may be because doctors have limited time to estimate mild physical discomfort during high-intensity work, leading to gradual HD worsening before the disease is taken seriously, and conservative treatment instead of surgery is usually selected after diagnosis. In the past, few studies examined doctors for medical condition. Doctors are both therapists and patients. Education of doctors is necessary, e.g., about the prevention, diagnosis and treatment of HD and other common diseases.

What’s more, this study also showed huge unmet needs concerning disease education. The HD prevalence in the well-educated population examined further demonstrated HD morbidity in this high-risk population and the limited knowledge of this disease, let alone the general population. The current treatment landscape and unfavorable outcome demand increased advocacy on guideline recommendation in the whole society to reduce the disease burden and improve the quality of life. There were limitations inherent to the online survey methodology that need to be considered when interpreting the results. These data were mostly from doctor-reported outcomes, and doctor selection bias, symptoms/signs recognition bias and recall bias need to be considered. However, some logistics errors have been modified by machine check, the questionnaire could be optimized further, and new techniques may be used to avoid potential bias in the future.

## Conclusion

OASIS is a large-scale web-based survey to examine the prevalence, awareness, diagnosis and treatment of HD in doctors in big cities of China. The results showed that HD prevalence in the Chinese doctor population was higher than that of the general population. However, doctors are mostly diagnosed and treated based on personal knowledge, lacking standardized treatment guideline, which affects the treatment effect. We urgently need to strengthen the education of doctors in HD to detect high-risk factors early for preventing HD occurrence, and to encourage patients to consult a doctor in time after the occurrence of HD symptoms. There is a gap between HD’s clinical practice and guideline recommendations, such as late initiation of and inadequate oral drug therapy. This may be one of the reasons for unsatisfactory therapeutic effect and high recurrence rate. Education of doctors in treatment guidelines needs to be strengthened, and standardized diagnosis and treatment plans suitable for HD patients are also required.

### Supplementary Information

**Supplementary material 1. **

## Data Availability

Please contact the corresponding author for data requests.
