# Supplementary material for: Hemorrhoidal disease among doctors from grade-A tertiary hospitals in big cities of China: results from web-based doctors as patients survey
Source: BMC Gastroenterol. 2024 Mar 13;24:103. doi: 10.1186/s12876-024-03166-2 (PMC10935840; doi:10.1186/s12876-024-03166-2)
Supplement: Supplementary file 1 — Supplementary material 1. [file 12876_2024_3166_MOESM1_ESM.pdf]

Contents

Screening part 1
HD Main Questionnaire 4
General information 10

Dear doctor,

We know that clinicians in long-term overwhelmed, will also suffer from chronic venous disease such as hemorrhoids or lower limb varicose veins, to understand current situation for doctor as patient with chronic venous disease, Medlive (http://www.medlive.cn/) launched a chronic venous disease diagnosis and treatment of clinical doctors in medical research activities, whether you have the disease can participate in answer, Thank you very much!
First, we will ask you a few questions to confirm which disease category you are answering.

Screening part

Q1 Please select your current main practice province/city [single-choice]

Beijing	○
Shanghai	○
Tianjin	○
Chongqing	○
Heilongjiang Province	○
JiLin Province	○
Liaoning Province	○
Inner Mongolia	○
Hebei Province	○
Henan Province	○
Shandong Province	○
Shanxi Province	○
Anhui Province	○
Jiangsu Province	○
Zhejiang Province	○
Jiangxi Province	○
Hunan Province	○
Hubei Province	○
Fujian Province	○
Guangdong Province	○

Hainan Province	○
Guangxi Province	○
Guizhou Province	○
Yunnan Province	○
Sichuan Province	○
Shaanxi Province	○
Ningxia Province	○
Gansu Province	○
Qinghai Province	○
Xinjiang	○
Xizang	○

Q2 Please select the level of your hospital [single-choice]

Grade-A tertiary	○	
Grade-B tertiary	○	Termination
Grade-C tertiary	○	Termination
Other	○	Termination

Q3 Please select your professional title [single-choice]

Chief Physician	○	
Associate chief physician	○	
Physician in Charge	○	
Resident physicians	○	Termination
Other	○	Termination

Q4 Please select your current main practice department [single-choice]

1	General internal medicine	1	
2	Department of Cardiology		
3	Department of Respiratory Medicine		
4	Department of Gastroenterology		
5	Department of Neurology		
6	Medical Oncology		
7	Endocrinology department/infectious department/nephrology and other internal medicine departments		
8	General surgery	2	
9	Department of Orthopedics		
10	Urology		
11	Obstetrics and gynecology		
12	Hepatobiliary surgery		
13	Breast surgery/neurosurgery/vascular surgery and other surgery		
14	Other Departments		Termination

Q5 Please select whether you now have the following diseases? [can be multiple-choice]

1	Hemorrhoidal disease	
2	Chronic venous disease (varicose veins of lower limb)	
3	Sleep disorders	
4	Lumbar/ cervical spine disease	
5	Hypertension.	
6	Chronic gastritis/gastric ulcer	
7	Other (please fill in)	

**If Q5=1**	**HD main questionnaire**
**if Q5≠1 and Q4=1-7**	**General information**

HD Main Questionnaire

Part One

Q1 How did you diagnose hemorrhoids? [single-choice]

1	Self-diagnosis	○
2	Diagnosis by general surgeon/anorectal specialist	○
3	By physical examination	○
4	Others (please fill out)	__

Q2 How many years have you been suffering from hemorrhoids? (If less than one year, please fill in "1") please fill in ____ years [fill in the blank] ———year(s)

Q3 The percentage of time you suffer from hemorrhoids per month is [single-choice]_

＜20%	○
20%-40%	○
40%-60%	○
60%-80%	○
＞80%	○

Q4 Which grade of hemorrhoids are you suffering from? [single-choice]

1	Grade 1: The main symptom is a small amount of hematochezia, no prolapse.	○
2	Grade 2: Hematochezia, prolapse during defecation, spontaneous reduction.	○
3	Grade 3: More hematochezia, prolapse during defecation, and requires manual reduction. After manual reduction, without prolapse again.	○
4	Grade 4: Severe hematochezia (maybe), and chronically prolapsed. Even after manual reduction, they will prolapse after a little movement.	○
5	Others (please fill out)	__

Q5 Do you know: a hemorrhoid is one of venous diseases, and more than half of patients with hemorrhoids were also complicated with chronic venous diseases (commonly known as varicose veins)? [single-choice]

1	Yes	○
2	No	○

Q6 In your opinion, which symptoms caused by hemorrhoids trouble you most? [can be multiple-choice]

1	Bleeding	□
2	Pain	□

3	Itching	□
4	Tenesmus	□
5	Anal dampness/effusion	□
5	Others (please fill out)	□

Q7 **How do you think the impact of hemorrhoids on your quality of life? [1-10 points] 1 point means almost no impact, 10 means great impact, the higher the score, the greater the impact on the quality of life**

Almost no impact ←→ Great impact

Q8 **What treatment methods have you used after the diagnosis of hemorrhoids? [can be multiple-choice]**

1	General treatment (intake more fruits and vegetables, drink more water, change the poor habits of defecation, and keep bowels open. Avoid prolonged sitting or standing, exercise properly, and warm hip bath (potassium permanganate can be used) before sleep, etc.)	□
2	Topical treatment (suppositories, ointments and lotions)	□
3	Oral drug	□
4	Injection therapy	□
5	Physical therapy	□
6	Rubber band ligation	□
7	Surgery	□
8	Infrared	□
9	Sclerotherapy	□
10	Others (please fill out)	___

Q9 Is it effective after the above treatment?

1	Effective without recurrence	○
2	Effective but with recurrence	○
3	Ineffective	○

Q10 **When do you think oral drugs should be taken? (Can choose up to 3 items) [can be multiple-choice]**

1	When the symptoms are severe and do not need to have used external drugs	□
2	Try external drugs first, and only use it after external drugs ineffective	□
3	Preoperative management of acute symptoms	□
4	Long-term use to prevent recurrence of hemorrhoids	□
5	Assist postoperative rapid recovery	□

Q11 **What oral drugs for hemorrhoids have you taken or are you taking? [can be multiple-choice]**

1	Alvenor (Citrus flavonoids Tablets - purified micronized flavonoids; former name Diosamine)	□
2	Aescuven forte (Aescuven forte Tablets)	□
3	SETUS-M (Melilotus Extract Tablets)	□
4	Darentang (Huaijiao Pills)	□
5	Xunkang (Zhisuning Tablets)	□
6	Junhong (Shuzhi Pills)	□
7	Dinghe (Huazhiling Tablets)	□
8	Jimin (Zhiyanxiao Granules)	□
9	Doxium (Calcium Dobesilate Capsules)	□
10	Aspirin Enteric-coated Tablets	□
11	Pletaal (Cilostazol Tablets)	□
12	Lunan (Mailuo Shutong Granules)	□
13	Yaodu (Dahuoluo Capsules)	□
14	Getai (Diosmin Tablets)	□
15	Mayinglong (Diosmin Tablets)	□
16	Venostan (Horse chestnut seed extract Tablets)	□
17	Oukai (Sodium Aescinate Tablets)	□
19	Others (please fill out)	□

Q12 **How satisfied are you with the drugs you have taken or are taking to treat hemorrhoids? [can be multiple-choice] only show the drugs selected in A11**

1 point=very unsatisfied, 10 points=very satisfied, the higher the score, the higher the satisfaction

		1	2	3	4	5	6	7	8	9	10
1	Alvenor (Citrus flavonoids Tablets - purified micronized flavonoids; former name Diosamine)	◎	◎	◎	◎	◎	◎	◎	◎	◎	◎
2	Aescuven forte (Aescuven forte Tablets)	◎	◎	◎	◎	◎	◎	◎	◎	◎	◎
3	SETUS-M (Melilotus Extract Tablets)	◎	◎	◎	◎	◎	◎	◎	◎	◎	◎
4	Darentang (Huaijiao Pills)	◎	◎	◎	◎	◎	◎	◎	◎	◎	◎
5	Xunkang (Zhisuning Tablets)	◎	◎	◎	◎	◎	◎	◎	◎	◎	◎
6	Junhong (Shuzhi Pills)	◎	◎	◎	◎	◎	◎	◎	◎	◎	◎

7	Dinghe (Huazhiling Tablets)	◎	◎	◎	◎	◎	◎	◎	◎	◎	◎
8	Jimin (Zhiyanxiao Granules)	◎	◎	◎	◎	◎	◎	◎	◎	◎	◎
9	Doxium (Calcium Dobesilate Capsules)	◎	◎	◎	◎	◎	◎	◎	◎	◎	◎
10	Aspirin Enteric-coated Tablets	◎	◎	◎	◎	◎	◎	◎	◎	◎	◎
11	Pletaal (Cilostazol Tablets)	◎	◎	◎	◎	◎	◎	◎	◎	◎	◎
12	Lunan (Mailuo Shutong Granules)	◎	◎	◎	◎	◎	◎	◎	◎	◎	◎
13	Yaodu (Dahuoluo Capsules)	◎	◎	◎	◎	◎	◎	◎	◎	◎	◎
14	Getai (Diosmin Tablets)	◎	◎	◎	◎	◎	◎	◎	◎	◎	◎
15	Mayinglong (Diosmin Tablets)	◎	◎	◎	◎	◎	◎	◎	◎	◎	◎
16	Venostan (Horse chestnut seed extract Tablets)	◎	◎	◎	◎	◎	◎	◎	◎	◎	◎
17	Oukai (Sodium Aescinate Tablets)	◎	◎	◎	◎	◎	◎	◎	◎	◎	◎
19	Others (please fill out)	◎	◎	◎	◎	◎	◎	◎	◎	◎	◎

Q13 **How did you choose these oral drugs? [can be multiple-choice] only show the drugs selected in Q11**

1	Doctor's prescription	□
2	Recommended by a pharmacist	□
3	Recommended by relatives, friends or other patients	□
4	Check on the Internet by oneself	□
5	Advertisement	□

Part two

Q1 **Which of the following factors do you think are most important in choosing an oral drug (Can choose up to 3 items) [multiple-choice]**

(Vertical options are arranged randomly)

1	Rapid onset, changes in symptoms can be felt within 2 days	□
2	Effectively improve symptoms such as bleeding, pain, and increased secretion	□
3	Reduce hemorrhoids recurrence	□
4	Extracted from natural plants, high safety	□

5	Can be used in combination with surgery to quickly control acute symptoms before surgery and accelerate postoperative recovery	□
6	More effective in combination with external drugs	□
7	The mechanism of action hits the core of the disease-venous inflammation	□

Q2 **Please score the following commonly used hemorrhoid drugs according to your understanding,** *1 point = totally disagree, 7 points = totally agree, the higher the score, the higher the degree of agreement. Add the "I don't know" option*

	(Vertical options are arranged randomly)	Alvenor (Citrus flavonoids Tablets - purified micronized flavonoids; former name Diosamine)	Aescuven forte (Aescuven forte Tablets)	SETUS-M (Melilotus Extract Tablets)	Darentang (Huaijiao Pills)	Xunkang (Zhisuning Tablets)	Junhong (Shuzhi Pills)	Dinghe (Huazhiling Tablets)	Jimin (Zhiyanxiao Granules)	Doxium (Calcium Dobesilate Capsules)
1	Rapid onset, changes in symptoms can be felt within 2 days	▿	▿	▿	▿	▿	▿	▿	▿	▿
2	Effectively improve symptoms such as bleeding, pain, and increased secretion	▿	▿	▿	▿	▿	▿	▿	▿	▿
3	Reduce hemorrhoids recurrence	▿	▿	▿	▿	▿	▿	▿	▿	▿
4	Extracted from natural plants, high safety	▿	▿	▿	▿	▿	▿	▿	▿	▿
5	Can be used in combination with surgery to quickly control acute symptoms	▿	▿	▿	▿	▿	▿	▿	▿	▿

	before surgery and accelerate postoperative recovery									
6	More effective in combination with external drugs	▽	▽	▽	▽	▽	▽	▽	▽	▽
7	The mechanism of action hits the core of the disease-venous inflammation	▽	▽	▽	▽	▽	▽	▽	▽	▽

	(Vertical options are arranged randomly)	Aspirin Enteric-coated Tablets	Pletaal (Cilostazol Tablets)	Lunan (Mailuo Shutong Granules)	Yaodu (Dahuoluo Capsules)	Getai (Diosmin Tablets)	Mayinglong (Diosmin Tablets)	Venostan (Horse chestnut seed extract Tablets)	Oukai (Sodium Aescinate Tablets)
1	Rapid onset, changes in symptoms can be felt within 2 days	▽	▽	▽	▽	▽	▽	▽	▽
2	Effectively improve symptoms such as bleeding, pain, and increased secretion	▽	▽	▽	▽	▽	▽	▽	▽
3	Reduce hemorrhoids recurrence	▽	▽	▽	▽	▽	▽	▽	▽
4	Extracted from natural plants, high safety	▽	▽	▽	▽	▽	▽	▽	▽
5	Can be used in combination with surgery to quickly control acute	▽	▽	▽	▽	▽	▽	▽	▽

	symptoms before surgery and accelerate postoperative recovery								
6	More effective in combination with external drugs	▽	▽	▽	▽	▽	▽	▽	▽
7	The mechanism of action hits the core of the disease-venous inflammation	▽	▽	▽	▽	▽	▽	▽	▽

Q3 **Do you know Alvenor (Citrus flavonoids Tablets - purified micronized flavonoids; former name Diosamine) has the following characteristics?** [**can be multiple-choice**]

1	Rapid onset, pain significantly relieved on the second day	□
2	Unique composition (5 active ingredients including diosmin), the effect is stronger than a single ingredient; the micronization process, make the absorption efficiency be nearly 2 times that of non-micronized diosmin	□
3	Act directly on the core of the disease, powerful intravenous anti-inflammatory and protective effects	□
4	Prevent hemorrhoids recurrence	□
5	Extracted from pure natural citrus, and safe	□
6	I don't know	□

General information

Q1 Do you know that other doctors in your department or relatives and friends around you had or are suffering from hemorrhoids? **[single-choice]**

1	Yes	○
2	No	○
3	I don’ t know	○

Q2 **Do you know: nearly half of Chinese adults suffer from hemorrhoids? [single-choice]**

1	Yes	○
2	No	○

Q3 **Do you know: a hemorrhoid is one of venous diseases, and more than half of patients with hemorrhoids were also complicated with chronic venous diseases (commonly**

known as varicose veins) [single-choice]

1	Yes	○
2	No	○

Q4 **If you have hemorrhoids, which treatment are you most likely to choose? [single-choice]**

1	General treatment (intake more fruits and vegetables, drink more water, change the poor habits of defecation, and keep bowels open. Avoid prolonged sitting or standing, exercise properly, and warm hip bath (potassium permanganate can be used) before sleep, etc.)	□
2	Topical treatment (suppositories, ointments and lotions)	□
3	Oral drug	□
4	Injection therapy	□
5	Physical therapy	□
6	Rubber band ligation	□
7	Surgery	□
8	Infrared	□
9	Sclerotherapy	□
10	Others (please fill out)	□

Q5 **As far as you know, which of the following oral drugs can be used to treat hemorrhoids? [can be multiple-choice]**

1	Alvenor (Citrus flavonoids Tablets - purified micronized flavonoids; former name Diosamine)	□
2	Aescuven forte (Aescuven forte Tablets)	□
3	SETUS-M (Melilotus Extract Tablets)	□
4	Darentang (Huaijiao Pills)	□
5	Xunkang (Zhisuning Tablets)	□
6	Junhong (Shuzhi Pills)	□
7	Dinghe (Huazhiling Tablets)	□
8	Jimin (Zhiyanxiao Granules)	□
9	Doxium (Calcium Dobesilate Capsules)	□
10	Aspirin Enteric-coated Tablets	□
11	Pletaal (Cilostazol Tablets)	□
12	Lunan (Mailuo Shutong Granules)	□
13	Yaodu (Dahuoluo Capsules)	□
14	Getai (Diosmin Tablets)	□
15	Mayinglong (Diosmin Tablets)	□
16	Venostan (Horse chestnut seed extract Tablets)	□
17	Oukai (Sodium Aescinate Tablets)	□
18	Others (please fill out)	□
19	Never heard of all above, unable to choose	□

Q6 **Do you know Alvenor (Citrus flavonoids Tablets - purified micronized flavonoids; former**

	**name Diosmin) has the following characteristics? [can be multiple-choice]**	
1	Rapid onset, pain significantly relieved on the second day	□
2	Unique composition (5 active ingredients including diosmin), the effect is stronger than a single ingredient; the micronization process, make the absorption efficiency be nearly 2 times that of non-micronized diosmin	□
3	Act directly on the core of the disease, powerful intravenous anti-inflammatory and protective effects	□
4	Prevent hemorrhoids recurrence	□
5	Extracted from pure natural citrus, and safe	
6	I don't know	□

Q7	**In the past six months, how many hours did you spend in outpatient clinics per week on average? [single-choice]**	
1	＜10 h	○
2	10-20 h	○
3	20-30 h	○
4	30-40 h	○
5	＞40 h	○

Q8	**What percentage of time do you spend in the following state in your daily work? (including outpatient and inpatient) [fill in the blank]**	
1	Standing	____%
2	Sitting	____%
3	Walking	____%
		Total 100%, showing the surplus Fill in any two options and automatically calculate the mode of the last ratio

Q9	**You defecate___times a day, or defecate once every_____days? [fill in the blank]**	≥0

Q10	**Which of the following types of stools do you usually have? [single-choice]**	
1	**Figure 1**	○
2	**Figure 2**	○
3	**Figure 3**	○
4	**Figure 4**	○
5	**Figure 5**	○
6	**Figure 6**	○
7	**Figure 7**	○

Picture shows the type of stool

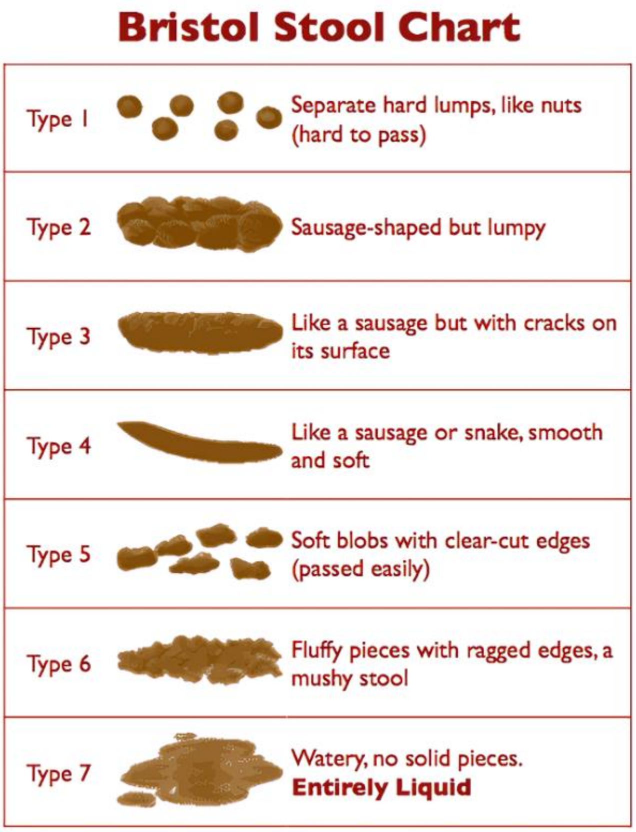

Q11 **How long does it usually take you to defecate? [single-choice]**

1	**＜5 min**	○
2	**5-10 min**	○
3	**10-20 mim**	○
4	**20-30 min**	○
5	**> 30 min**	○

Q12 **What is your age? Please fill in____ one full year of life** ———years *22-70*

Q13 **What is your gender? [single-choice]**

1	Male	○
2	Female	○

Q14 **ASK if D7=2** How many times are your pregnancies (____)**[fill in the blank]**

1	2 or more	○
2	1	○
3	0	○

Q15 Do you smoke? [**single-choice**]

1	Yes	○
2	No	○

Q16 **ASK IF D9=1** Normally, how many cigarettes do you smoke a day? [single-choice]

1	＜Half pack (＜10 cigarettes/day)	○
2	Half to one pack (10-20 cigarettes/day)	○
3	One to two packs (20-40 cigarettes/day)	○
4	＞Two packs (＞40 cigarettes/day)	○
5	I smoke an electronic cigarette	○

Q17 What are your height and weight? **[fill in the blank]**

Height___(m) Weight___(kg)	
According to your height and weight, your BMI index is calculated as: _____	Calculation formula: body mass index (BMI) = weight (kg) ÷ height² (m²)

Q18 How much time do you spend on exercise per week?-[**single-choice**]

1	＜0.5 h	○
2	0.5-1 h	○
3	1-2 h	○
4	2-4 h	○
5	＞4 h	○

Q19 **Do you often troubled by constipation (decrease in frequency of defecation, dry and hard stools and difficulty in defecating. Defecation less than 3 times per week)?** [**single-choice**]

1 Troubled by constipation in the last 3 months ○
2 Used to be troubled by constipation ○
3 Never constipated ○

Q20 **Where would you like to learn about the treatment of hemorrhoids? [can be multiple-choice]**

1	WeChat Subscription	□
2	Microblog	□
3	Zhihu	□
4	Tik Tok	□
5	Medical lectures (Continuing education training courses, etc.)	□

6	Dissemination of publicity materials	□
7	Related guidelines	□
8	Medical representative visits	□
9	Professional medical information platform (such as Medlive.cn)	□
10	Others (please fill out)	□
